# Extended Co-Expression of Inhibitory Receptors by Human CD8 T-Cells Depending on Differentiation, Antigen-Specificity and Anatomical Localization

**DOI:** 10.1371/journal.pone.0030852

**Published:** 2012-02-08

**Authors:** Lukas Baitsch, Amandine Legat, Leticia Barba, Silvia A. Fuertes Marraco, Jean-Paul Rivals, Petra Baumgaertner, Céline Christiansen-Jucht, Hanifa Bouzourene, Donata Rimoldi, Hanspeter Pircher, Nathalie Rufer, Maurice Matter, Olivier Michielin, Daniel E. Speiser

**Affiliations:** 1 Clinical Tumor Biology and Immunotherapy Unit, Ludwig Center, University of Lausanne, Lausanne, Switzerland; 2 University Hospital Center, University of Lausanne, Lausanne, Switzerland; 3 Department of Immunology, Institute of Medical Microbiology and Hygiene, University of Freiburg, Freiburg, Germany; 4 Service of Radiation Oncology, Lausanne University Hospital, Lausanne, Switzerland; Wayne State University, United States of America

## Abstract

Inhibitory receptors mediate CD8 T-cell hyporesponsiveness against cancer and infectious diseases. PD-1 and CTLA-4 have been extensively studied, and blocking antibodies have already shown clinical benefit for cancer patients. Only little is known on extended co-expression of inhibitory receptors and their ligands. Here we analyzed the expression of eight inhibitory receptors by tumor-antigen specific CD8 T-cells. We found that the majority of effector T-cells simultaneously expressed four or more of the inhibitory receptors BTLA, TIM-3, LAG-3, KRLG-1, 2B4, CD160, PD-1 and CTLA-4. There were major differences depending on antigen-specificity, differentiation and anatomical localization of T-cells. On the other hand, naive T-cells were only single or double positive for BTLA and TIM-3. Extended co-expression is likely relevant for effector T-cells, as we found expression of multiple ligands in metastatic lesions of melanoma patients. Together, our data suggest that naive T-cells are primarily regulated by BTLA and TIM-3, whereas effector cells interact via larger numbers of inhibitory receptors. Blocking multiple inhibitory receptors simultaneously or sequentially may improve T-cell based therapies, but further studies are necessary to clarify the role of each receptor-ligand pair.

## Introduction

Upon activation, T-cells upregulate several hundred genes required for proper proliferation, differentiation and function of effector and memory T-cells [1], [2], [3]. In parallel to activatory receptors and pathways, T-cells also express several inhibitory receptors [4], [5]. These receptors mediate T-cell hyporesponsiveness and thus play a central role in preventing overwhelming T-cell activation, immune pathology and autoimmunity, but also destruction of cancer cells [6], [7], [8]. Generally, these receptors are upregulated with progressive T-cell differentiation, with the notable exception of BTLA, which is high on naive cells but downregulated in memory and effector cells [9], [10]. Therapeutic blockade of inhibitory receptors (e.g. by using antibodies) can augment T-cell functionality [11], which is even more pronounced when two inhibitory receptors are blocked simultaneously [8], [12], [13], [14], [15], [16]. Blocking of individual inhibitory receptors has now become a novel approach to treat cancer patients. In March 2011, the FDA has approved the monoclonal anti-CTLA-4 antibody Ipilimumab for melanoma patients [17], [18], [19]. Another anti-CTLA-4 antibody (Tremilimumab) [20] and anti-PD-1 antibodies are in clinical development [21].

Several further inhibitory receptors (CD160, KLRG-1, TIM-3, 2B4, BTLA and LAG-3) have been studied individually [9], [22], [23], [24], [25], [26]. In contrast, their co-expression has not yet been investigated in greater detail in cancer patients. Multiple inhibitory receptors have been implicated in the induction of T-cell exhaustion, a state of T-cell hyporesponsiveness that is frequently found in chronic viral infections [5], [8], [11], [27]. We have recently shown that functional T-cell deficiency in melanoma metastases is associated with gene expression characteristics of exhausted T-cells [1], with significant similarity to chronic/protracted viral infection [4]. In accordance, individuals with cancer show enhanced expression of inhibitory receptors [28], [29].

Here we determined expression patterns by analyzing eight inhibitory receptors on tumor-antigen specific CD8 T-cells. We found that apart from BTLA and TIM-3 these receptors were mostly undetectable on naive T-cells, but upregulated following priming and differentiation. In addition, we found altered inhibitory receptor expression patterns in CD8 T-cells analyzed directly after isolation from melanoma metastases. In parallel, we studied the ligands of these inhibitory receptors, and found that many of them are expressed by melanoma cells and/or in the tumor stroma. The data suggest that inhibition of tumor-specific CD8 T-cells is mediated by multiple inhibitory receptors and depends on antigen-specificity, differentiation and anatomical localization of T-cells.

## Materials and Methods

### Ethics statement

The clinical studies were designed and conducted according to the relevant regulatory standards, and approved by the ethical commission of the University of Lausanne and by Swissmedic. Blood and tissue were obtained upon written informed patient consent.

### Clinical trials

Vaccinations were done in the context of three consecutive clinical trials of the Ludwig Institute for Cancer Research [30], [31], [32] with similar study designs, and the same treatment schedule and primary endpoint, i.e. induction of cancer-specific T-cell responses. HLA-A*0201^+^ patients with stage III/IV metastatic melanoma received multiple monthly low-dose vaccinations s.c. with 100 µg Melan-A/MART-1 peptide and with or without CpG-ODN (500 µg of the oligonucleotide PF-3512676/7909; provided by Pfizer/Coley Pharmaceutical Group, U.S.A.), emulsified in 300–600 µl IFA (Incomplete Freund's Adjvuant, i.e. Montanide ISA-51 provided by Seppic, France) as described previously [30], [32] or 3 times 500 µg peptides (NY-ESO-1, MAGE-A10 and Melan-A) emulsified in IFA with or without CpG-ODN [31].

### Blood and lymph node cells

Peripheral blood was obtained from patients, and from A2^+^ healthy donors through the University Blood Transfusion Center of Lausanne, Switzerland. Metastatic lymph nodes were obtained from melanoma patients after 7±2 vaccinations, the last one at a mean of 79 days before surgery. T-cells from tumor-infiltrated lymph nodes (TILN) were prepared after finely mincing surgery specimens. Mononuclear cells were purified by density gradient using Lymphoprep (Axis-Shieldy) and immediately cryopreserved in RPMI 1640 supplemented with 40% FCS and 10% DMSO.

### Flow cytometry

Cells were analyzed directly ex vivo, i.e. without prior culturing. Cells were stained using A2/EBV BMLF1_280–288_ (GLCTLVAML) tetramer, A2/CMV pp65_495–503_ (NLVPMVATV) tetramer, A2/Melan-A/MART-1_26–35_A27L (ELAGIGILTV) tetramer, A2/NY-ESO-1_157–165_ (SLLMWIITQA) tetramer and A2/MAGE-A10_254–262_ (GLYDGMEHL) tetramer. Melan-A-specific tetramers were labeled with APC-eFluor®780 (eBioscience), EBV- and NY-ESO-1-specific tetramers were labeled with PE-TexasRed (BD Pharmingen) and CMV- and MAGE-A10-specific tetramers were labeled with both APC-eFluor®780 and PE-TexasRed allowing for individual analysis of T-cells specific for three epitopes in a single sample. Inhibitory receptors were stained as described previously [1]. Briefly, surface staining was performed on PBMC magnetically enriched for CD8 T-cells (Invitrogen, purity >95%), with antibodies specific for CD8-PacificBlue, CCR7-PC7, CD45RA-APC-A700 and four inhibitory receptors for “staining 1”, i.e. KLRG-1-A488 (provided by H. Pircher), TIM-3-PE (R&D Systems), PD-1-PerCP-eFl710 (eBioscience) and CD160-A647 (eBioscience), or three inhibitory receptors for “staining 2”, i.e. LAG-3-FITC (Alexis Biochemicals), BTLA-PE (BD) and 2B4-PE-Cy5 (BioLegend). Staining 2 was completed with CTLA-4-APC (BD) in FACS buffer with 0.1% saponin, after the cells had been fixed for 30 minutes at room temperature (1% formaldehyde-buffer). LIVE/DEAD-Fixable-Aqua (Invitrogen) was used as a dead cell exclusion marker and appropriate isotype controls were used to define negative populations. Because of limitations of available cell numbers, not all samples could be analyzed by both staining 1 and staining 2. The gating strategy is shown in *Figure S1A*. Magnetic enrichment for CD8^+^ cells in addition with careful gating on the CD8^+^ cells allowed minimization of contaminations by other cell populations such as NK and NKT cells.

Melanoma cell lines were generated as described [33] and grown in RPMI 1640 medium supplemented with 10% FCS. Surface stainings were performed using antibodies specific for CD48-FITC (BioLegend), CD80-PE-Cy7 (BD), CD86-APC-Alexa700 (BD), HVEM-PE (BioLegend), PD-L2-APC (BioLegend), PD-L1-PE-Cy7 (BD), E-cadherin-APC (BioLegend), HLA-II-FITC (Abcam). In addition, a PE-coupled galectin-9 specific antibody (BioLegend) was used for intracellular staining.

Data was acquired on a Gallios Flow Cytometer (Beckman Coulter) and analyzed using FlowJo 9.1 (TreeStar). Analysis of co-expression of inhibitory receptors was done with SPICE software version 5.2 [34].

### Immunohistochemistry

Consecutive paraffin sections (4 µM thick) were cut and mounted on electrostatically precharged slides (Superfrost Plus, Menzel-Gläser). The sections were deparaffinized in xylene and rehydrated through graded alcohols. After endogenous peroxidase quenching (0.3% H_2_O_2_ in distilled water for 5 minutes), antigens were retrieved by boiling the sections in 1 mM EDTA solution, pH 9.0, in a pressure cooker for 3 minutes. Tissue sections were incubated for 1 hour at RT with an antibody specific for HLA-class II (Novus Biologicals), CD80 (Novus Biologicals), CD86 (Novus Biologicals), CD273 (Atlas Antibodies), CD48 (Abnova), galectin-9 (R&D Systems), or HVEM (Alexis Biochemicals). After washing, tissue sections were incubated for 30 minutes at RT with horseradish peroxidase–conjugated anti-rat, anti-mouse or anti-sheep IgG (Dako) and diaminobenzidine (Dako). Finally, the sections were counterstained with hematoxylin and dehydrated through graded alcohols and xylene. Immunohistological data of HVEM was already published in ref. [9] and is shown here for completion.

### Statistical calculations

Hierarchical clustering and principal component analysis (PCA) was performed using the program *R* version 2.13.0. For quantitative comparisons, Student's t-test (two-sample two-tailed comparison) or one-way ANOVA with Tukey post-test (multiple-sample comparison) were performed with Prism 5.0 unless otherwise noted. Co-expression pie charts were compared with each other using 10'000 permutations calculated with the software SPICE 5.2. p<0.05 was considered significant (* = p<0.05; ** = p<0.01; *** = p<0.001; ns = not significant).

## Results

### Inhibitory receptors are upregulated with CD8 T-cell differentiation

**Figure 1:**
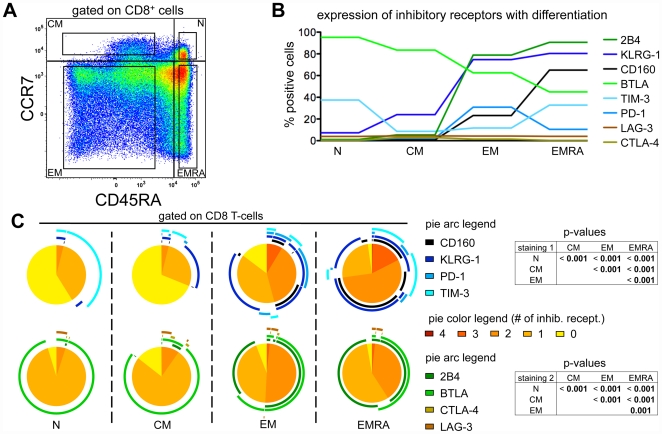
Expression profiles of inhibitory receptors with differentiation. (A) CD8 T-cell subsets were defined depending on expression of CCR7 and CD45RA, namely naive (N), central memory (CM), effector memory (EM) and effector memory RA^+^ (EMRA) cells. Gates used for inhibitory receptor analysis are shown in the four quadrants. (B) Mean values of inhibitory receptor expression in relation to the differentiation status. Individual values are shown in *Figure S1B*. n = 31 for “staining 1” (KLRG-1, TIM-3, PD-1 and CD160); n = 21 for “staining 2” (LAG-3, BTLA, 2B4 and CTLA-4); four samples of staining 1 were from healthy donors, the remaining from melanoma patients. (C) Co-expression of KLRG-1, TIM-3, PD-1 and CD160 (staining 1) and of LAG-3, BTLA, 2B4 and CTLA-4 (staining 2). Colors of the pie arcs depict the expression of individual inhibitory receptors, while the color in the pie depicts the number of co-expressed inhibitory receptors. p-values of the permutation test are shown in tables next to the corresponding pie charts. Co-expression was analyzed with SPICE 5.2.

By flow cytometry, we analyzed co-expression of the eight inhibitory receptors CD160, KLRG-1, PD-1, TIM-3 (“staining 1”) and 2B4, BTLA, CTLA-4 and LAG-3 (“staining 2”) depending on T-cell differentiation status. Peripheral blood mononuclear cells (PBMC) were stained with antibodies specific for these inhibitory receptors, as well as for CD8, CD45RA and CCR7. We gated on CD8 naive (N), central memory (CM), effector memory (EM) and effector memory CD45RA^+^ (EMRA) cells defined by CCR7 and CD45RA expression (*Figure 1A*). Naive cells were frequently BTLA positive and many of them also expressed TIM-3 (*Figure 1B* *and S1B*). All other inhibitory receptors were upregulated along with progressive differentiation. While EMRA cells were frequently positive for 2B4, KLRG-1 and CD160, they expressed less PD-1 and BTLA than EM cells. The expression levels of LAG-3 and CTLA-4 were very low in all subpopulations of CD8 T-cells. Analysis of co-expression patterns of inhibitory receptors revealed that not only the overall frequencies of inhibitory receptor expression increased with differentiation, but also the variability of co-expression of multiple inhibitory receptors (*Figure 1C*). While only negligible numbers of N and CM cells co-expressed two to four of the inhibitory receptors CD160, KLRG-1, PD-1 and TIM-3 (staining 1), roughly half the EM cells and three quarters of the EMRA cells did so (*Figure 1C* *, upper panels*). Co-expression of PD-1 and TIM-3 was observed in only a small portion of the EM cells while co-expression of TIM-3 and CD160 was present only in EMRA cells. A slightly different picture was observed for staining 2 (2B4, BTLA, CTLA-4 and LAG-3) (*Figure 1C* *, lower panels*). The highest percentage of co-expression of these inhibitory receptors was observed in EM cells, with roughly 50% of the cells positive for 2B4 and BTLA. While in naive cells BTLA was frequently expressed alone, its expression in EM and EMRA cells was almost exclusively together with 2B4.

Overall, these data show that inhibitory receptors are progressively upregulated with differentiation, with the remarkable exceptions of BTLA and TIM-3. This pattern of expression is in agreement with a recent report investigating the expression of 2B4, KLRG-1, CD160 and PD-1 together with CD127 and CD27 on HCV-specific CD8 T-cells [5].

### Co-expression of inhibitory receptors of tumor-specific T-cells is influenced by vaccination

**Figure 2:**
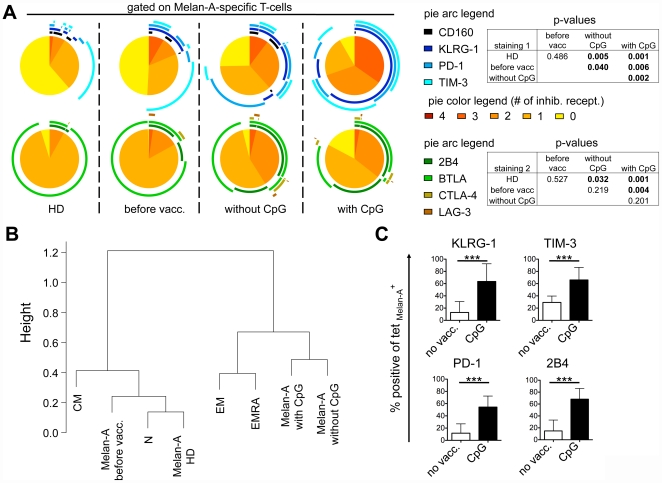
Inhibitory receptor expression by Melan-A specific CD8 T-cells depending on vaccination. (A) Co-expression of KLRG-1, TIM-3, PD-1 and CD160, and of LAG-3, BTLA, 2B4 and CTLA-4 by Melan-A specific CD8 T-cells. Blood samples from healthy donors (HD) or from patients before immunotherapy (before vacc.) or after peptide+IFA vaccination with or without CpG-ODN 7909 were enriched for CD8 T-cells using magnetic beads. Melan-A-specific CD8 T-cells were identified by staining with CD8-specific antibody and tetramer. Positivity for inhibitory receptors was defined respective to isotype controls. n = 4 for HD; n = 3 for before vacc.; n = 9 for after vaccination without CpG-ODN and n = 11 for after vaccination with CpG-ODN. Colors of the pie arcs depict the expression of individual inhibitory receptors, while the color in the pie depicts the number of co-expressed inhibitory receptors. Co-expression was analyzed with SPICE 5.2. p-values of the permutation test are shown in tables next to the corresponding pie charts. (B) Hierarchical clustering based on co-expression data of the eight inhibitory receptors shown in A, including the four differentiation subsets (N, CM, EM, EMRA) of total CD8 T-cells. (C) Mean expression and SD of four inhibitory receptors upregulated on Melan-A-specific T-cells with vaccination. Data from HD and from patients before vaccination were pooled for the group without vaccination (no vacc.). n = 7 for no vacc.; n = 9 for vaccination with CpG-ODN.

Despite their naive status, Melan-A specific T-cells can be detected ex vivo using tetramers in PBMC from healthy donors [35]. In melanoma patients, the frequency of these T-cells increases occasionally with disease progression and/or after vaccination without CpG-ODN. In sharp contrast, nearly all patients show strongly increased T-cell frequencies when CpG-ODNs are used as vaccine adjuvant [32]. We have previously shown that the type of vaccination impacts on the expression of BTLA [9] and that tumor-specific T-cells after vaccination express multiple inhibitory receptors [1]. To investigate expression patterns of specific inhibitory receptors, we first compared the naive Melan-A-specific cells from healthy donors to the total pool of naive T-cells (*Figure 1C* and *2A*). Most of the Melan-A-specific T-cells from healthy donors did not express inhibitory receptors, apart from BTLA and TIM-3. Therefore, they closely resembled total naive T-cells (*Figures 1C* and *2A*). Furthermore, Melan-A-specific T-cells in melanoma patients before vaccination also closely resembled total naive T-cells and Melan-A-specific T-cells from healthy donors (*Figure 2A*). This is likely due to the fact that in melanoma patients, spontaneously activated cells are usually infrequent and only weakly activated [30].

Subsequently, we extended the inhibitory receptor analysis to patients after vaccination with Melan-A peptide, with or without the addition of CpG-ODN 7909 to the vaccine formulation (*Figures 2A* *and S2*), and compared the data to the other two conditions, i.e. healthy donors and melanoma patients before vaccination. Interestingly, the four receptors KLRG-1, TIM-3, PD-1 and 2B4 were all significantly upregulated on Melan-A specific T-cells with vaccination, especially with CpG-ODN. On the contrary, BTLA expression was significantly downregulated as described previously [9]. Finally, CD160, LAG-3 and CTLA-4 were expressed at very low levels without significant differences between the four conditions. Regarding simultaneous co-expression, the Melan-A-specific cells were more often positive for two or more inhibitory receptors when CpG-ODN was used for vaccination as compared to “without CpG-ODN”. Especially the population triple positive for KLRG-1, PD-1 and TIM-3 made up more than 30% of all Melan-A-specific T-cells after vaccination with CpG-ODN. Even though technical issues prevented us from investigating the co-expression patterns between KLRG-1, PD-1 and TIM-3 (staining 1) on one side, and 2B4 and BTLA (staining 2) on the other side, it is clear from this analysis that at least some of those triple positive samples co-expressed BTLA and/or 2B4, making them quadruple positive or more.

Confirming these observations, hierarchical clustering based on the co-expression of the eight inhibitory receptors, showed that Melan-A-specific T-cells from healthy donors or from patients before vaccination were closely related to the total pool of naive CD8 T-cells (N), which changed significantly after vaccination where the Melan-A specific T-cells resembled EM and EMRA cells (*Figure 2B*). Strong upregulation of KLRG-1, TIM-3, PD-1 and 2B4 (*Figure 2C*), and concomitant downregulation of BTLA (*Figure S2*) was observed after vaccination, particularly when CpG-ODN was used as adjuvant, emphasizing that vaccination may have profound effects on inhibitory receptor expression.

### The pattern of inhibitory receptor expression by Melan-A-specific T-cells is similar to other self-specific T-cells but differs from virus-specific T-cells

**Figure 3:**
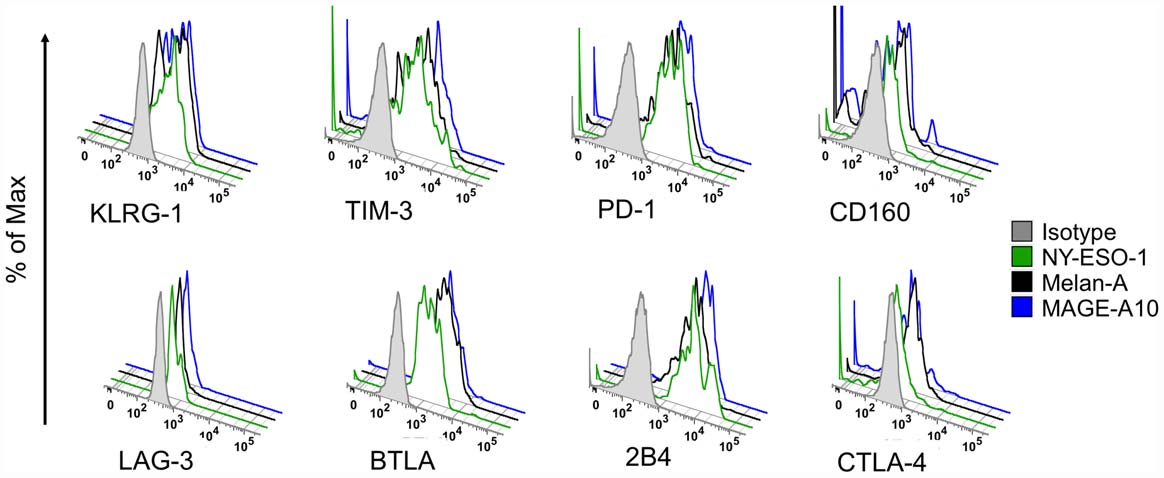
Expression of inhibitory receptors on self/tumor-specific T-cells. Expression of inhibitory receptors by Melan-A, NY-ESO-1 and MAGE-A10-specific T-cells from a representative patient (LAU 1169). CD8 T-cells were enriched using magnetic bead sorting. Melan-A- (black), NY-ESO-1- (green) and MAGE-A10- (blue) specific CD8 T-cells were identified by tetramer staining as described in the Materials and Methods section. An isotype control (grey) is shown as reference.

We have recently reported [1] that Melan-A-specific T-cells expressed different patterns of inhibitory receptors compared to virus-specific T-cells. Here we extended this analysis by including BTLA (*Figure S3A*) and investigating the co-expression patterns of Melan-A-, CMV- and EBV-specific T-cells. We observed that all three antigen-specific T-cell populations resembled EM and EMRA cells more than N and CM cells. However, tumor-specific T-cells differed more from EM and EMRA cells than the virus-specific cells both in a hierarchical clustering (*Figure S3B*) and in a principal component analysis (*Figure S3C*). This intermediate position of Melan-A-specific cells is mainly due to their expression of TIM-3 and virtual absence of CD160 (*Figure S3A* and reference [1]). Next, we investigated whether the peculiarities observed for Melan-A-specific T-cells was a general feature of self/tumor-specific T-cells. In the framework of our melanoma program, some patients were vaccinated simultaneously with three different short peptides derived from Melan-A, NY-ESO-1 and MAGE-A10 [31]. We could analyze four patients with ex vivo detectable CD8 T-cell responses against all three peptides. In contrast to the differing patterns observed between Melan-A- and virus-specific T-cells (*Figure S3A*), NY-ESO-1- and MAGE-A10-specific T-cells showed patterns comparable to Melan-A-specific T-cells, with no significant differences in any of the eight inhibitory receptors (*Figures 3* and *S4*). In addition, the Melan-A-specific T-cells from these four patients were highly similar to the Melan-A-specific T-cells from the first group analyzed (*Figures S3A* and *S4*). This observation suggests that the particular expression pattern observed in tumor-specific T-cells is either a general property of self/tumor-specific cells, or else induced by vaccination.

### Influence of the microenvironment on the expression of inhibitory receptors

**Figure 4:**
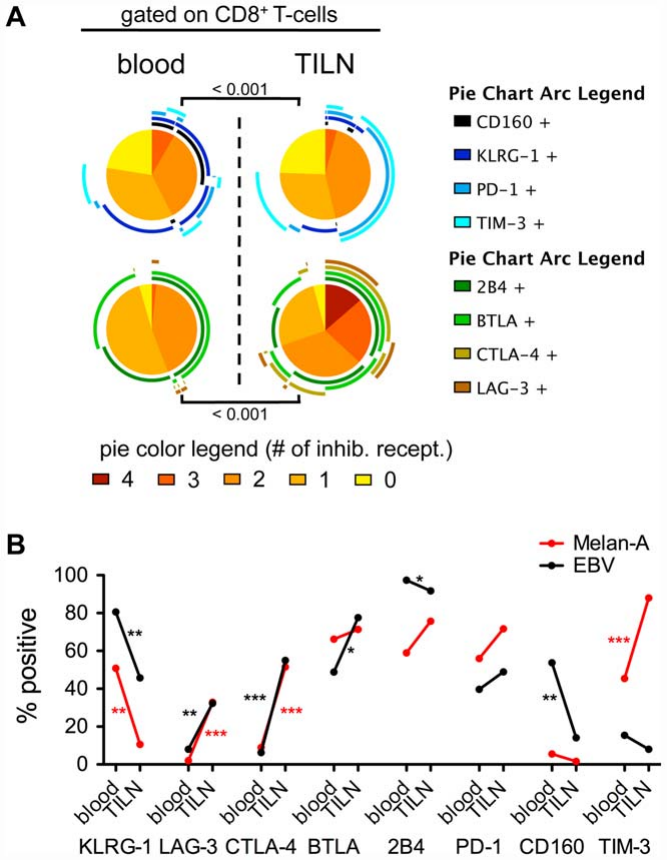
Expression of inhibitory receptors by CD8 T-cells derived from blood and tumor-infiltrated lymph nodes (TILNs). (A) Co-expression analysis of total CD8 T-cells. Colors of the pie arcs depict the expression of individual inhibitory receptors, while the color in the pie depicts the number of co-expressed inhibitory receptors. Co-expression was analyzed with SPICE 5.2. n = 9/8 (TILN) and 31/17 (blood) for staining 1 and staining 2 respectively. (B) Melan-A- (red) and EBV- (black) specific CD8 T-cells. Positivity for the inhibitory receptor was defined respective to isotype controls. Blood samples were from patients vaccinated either with CpG-ODN or without CpG-ODN. n = 20/21 (blood; Melan-A/EBV) and n = 9/6 (TILN; Melan-A/EBV) for staining 1; n = 23/24 (blood; Melan-A/EBV) and n = 8/5 (TILN; Melan-A/EBV) for staining 2 except BTLA, and n = 8/7 (TILN; Melan-A/EBV) for BTLA.

The tumor microenvironment may strongly influence protein expression, including inhibitory receptors [1]. Therefore, we compared T-cells from PBMC and tumor infiltrated lymph nodes (TILN). We observed an upregulation of LAG-3, CTLA-4 and TIM-3 and a downregulation of KLRG-1 and CD160 in total CD8 T-cells obtained from TILN compared to blood (*Figure 4A*). Since it is likely that many of those total CD8 T-cells from TILN are tumor specific, our data suggest that they frequently express inhibitory receptors such as CTLA-4, LAG-3, PD-1, TIM-3, BTLA and 2B4.

Tumor-specific and non-tumor-specific T-cells in TILN may be affected differently by the presence of tumor cells. Melan-A-specific T-cells from TILN expressed much less KLRG-1 but more TIM-3, LAG-3 and CTLA-4 than their counterparts from the blood (*Figure 4B* *, red and Figure S5A*). We could not observe significant differences in the expression of CD160, BTLA, 2B4 and PD-1, although TILN derived CD8 T-cells have been shown to express more PD-1 than in cells from the circulation [36]. Overall, Melan-A-specific cells in TILN had a higher expression of inhibitory receptors than cells from the blood. This observation fits with our recent finding that Melan-A-specific T-cells from TILN show an exhaustion profile, i.e. a gene expression profile matching exhausted T-cells, associated with impairment of cytokine production and enhanced expression of inhibitory receptors [1].

To study the impact of the lymph node microenvironment on non-tumor-specific T-cells, we analyzed EBV-specific T-cells from TILN (*Figure 4B* *, black* and *Figure S5B*). These cells should not (or less) be influenced by the presence of tumor cells in the metastatic lymph nodes, as they are not tumor-specific, whereas they may interact with normal lymph node cells. Interestingly, we found a modulation of inhibitory receptors on the surface of EBV-specific T-cells in TILN as compared to blood (*Figure 4B*), in part similar to Melan-A-specific T-cells. Specifically, we found a downregulation of KLRG-1 and CD160 in TILN. Also for 2B4, we observed a lower expression in TILN – the absolute difference was small but nevertheless significant. On the contrary, both LAG-3 and CTLA-4 were upregulated, mirroring Melan-A-specific T-cells. Notably and in contrast to Melan-A-specific T-cells, there was no change in the expression of TIM-3 on EBV-specific T-cells from TILN.

Overall, these results demonstrate a major influence of the microenvironment on the expression of several inhibitory receptors on total CD8 and on antigen-specific T-cells. Compared to the blood, KLRG-1 downregulation goes hand-in-hand with LAG-3 and CTLA-4 upregulation in TILN as compared to blood. Other changes in surface expression, for example the upregulation of TIM-3 in Melan-A-specific T-cells or the downregulation of CD160 in EBV-specific T-cells are likely antigen-specific effects.

### Several ligands for inhibitory receptors are expressed in melanoma lesions

**Figure 5:**
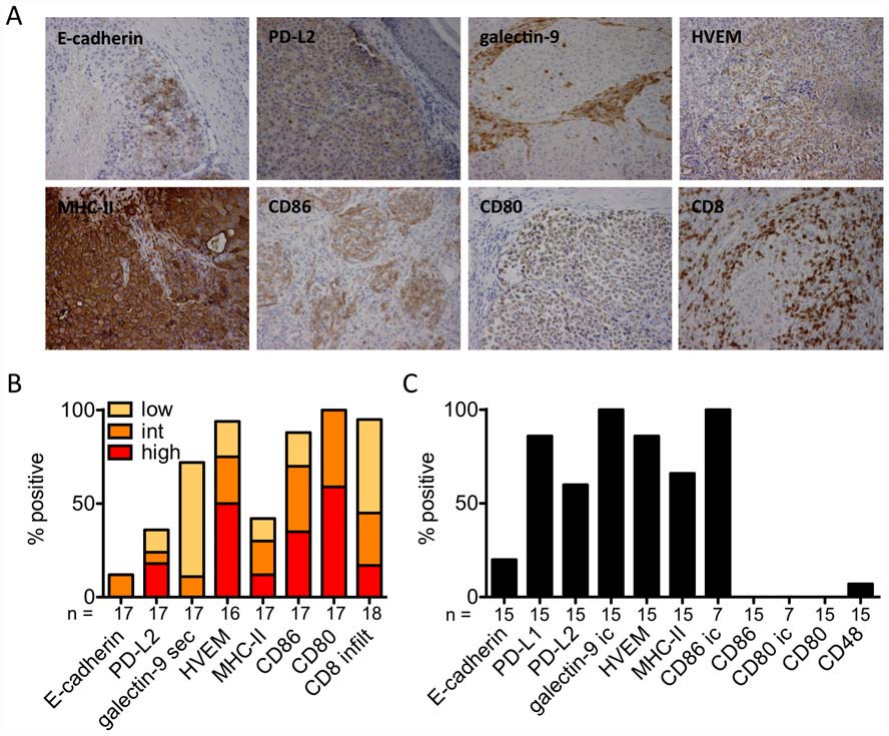
Expression of ligands of inhibitory receptors in melanoma metastases and by melanoma cell lines. (A,B) Paraffin-embedded tumor sections from 16 to 18 tumors were stained by immunohistochemistry for seven inhibitory receptors and CD8. (A) Representative stainings (magnification ×200) for each ligand investigated. (B) Summary of immunohistochemical stainings represented as percent of positive samples. Low (<10%), intermediate (int; 10–50%) and high (>50%) expression is indicated in a color scale. infilt: infiltration of CD8 T-cells in tumor cell nests; sec: secreted i.e. intra- and extracellular presence of galectin-9. (C) Summary of expression by melanoma cell lines on the surface or intracellular (ic) as percent of positive cell lines.

Binding of inhibitory receptors to their respective ligands (*Table S1*) is probably necessary for inhibiting T-cell function. To determine whether these ligands are expressed in melanoma metastases, we performed immunohistochemical analysis on 16 to 18 paraffin-embedded tumor sections (*Figure 5A*). With one exception, all tumors were infiltrated by CD8 T-cells (3/18 strongly and 14/18 moderately). Most tumors expressed intracellular CD80 (100%), HVEM (93%) and CD86 (88%) and 9/17 expressed at least one of the three ligands MHC class II, PD-L2 and galectin-9 (*Figure 5B*). In contrast, only 2/17 tumor sections showed expression of E-cadherin. Eight tumors were available for the analysis of the expression of all seven ligands. All of these lesions expressed at least three and 3/8 expressed at least four of the seven tested ligands. Tumor infiltrating lymphocytes not only interact with the tumor cells themselves, but also with the stroma surrounding the tumor. Hence, ligands of inhibitory receptors expressed in the tumor stroma may also have a negative impact on lymphocyte function. In the tumor microenvironment, we found cells expressing MHC class II and secreted galectin-9 in most of the cases, and CD80 and PD-L2 in some cases (*Figure 5B* and data not shown). In parallel, we analyzed 15 melanoma cell lines generated from metastatic tissue from melanoma patients by flow cytometry. Most cells showed surface expression of PD-L1, PD-L2, MHC II and HVEM but not of CD48 (*Figure 5C*). Confirming our results on paraffin sections, E-cadherin expression was infrequent on melanoma cell lines, in line with the known E-cadherin downregulation by metastatic tumor cells [37] and corresponding to the diminished expression of KLRG-1 by CD8 T-cells found in TILN (*Figure 4*). Seven cell lines were also analyzed for intracellular expression. In contrast to the paraffin-sections, all melanoma cell lines expressed intracellular galectin-9. 7/7 lines expressed intracellular CD86, however, none of them showed CD80 expression. Even though a large heterogeneity in the expression of inhibitory receptor ligands was observed in tumor sections and on melanoma cell lines, all samples expressed at least some of these ligands, suggesting that this type of negative regulation of T-cell function may often be relevant in the tumor microenvironment.

## Discussion

The extended upregulation of inhibitory receptors plays many roles in the control of activated T-cells and in the prevention of tissue damage by cytotoxic T-cells. Even though the signaling cascades downstream of inhibitory receptors are likely to overlap, their functions are not necessarily redundant. The fact that their expression is differently regulated depending on the T-cell activation status argues for distinct functions of different inhibitory receptors.

**Figure 6:**
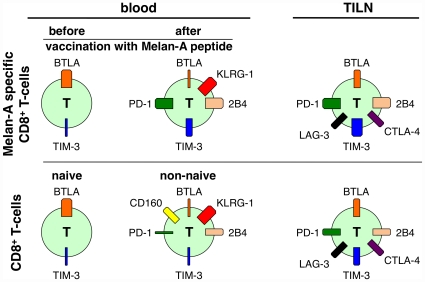
Schematic representation of inhibitory receptor co-expression according to differentiation status and physical location. Naive cells express BTLA and TIM-3. After peptide vaccination, Melan-A specific T-cells upregulate KLRG-1, 2B4, TIM-3 and PD-1, while they downregulate BTLA. Total CD8 T-cells upregulate similar inhibitory receptors, but less PD-1 and TIM-3. They also express CD160, which is not expressed by tumor-specific T-cells. In TILN, both total CD8 T-cells, which are to a large extent tumor-specific, and Melan-A specific T-cells downregulate KLRG-1 (and in total CD8 T-cells CD160) and concomitantly express more PD-1, LAG-3, TIM-3 and CTLA-4.

Interestingly, T-cell differentiation impacts differentially on the expression of individual inhibitory receptors. Only two of the eight studied inhibitory receptors, i.e. BTLA and TIM-3 were clearly detectable already at the naive stage (*Figure 6*). BTLA was highest in naive cells and lowest in EMRA cells, and TIM-3 was intermediate on both naive and EMRA cells and low on central- and effector memory cells. Many genes are modulated along T-cell differentiation [3], [38]. Apparently, inhibitory receptors play different roles depending on the differentiation status, and may also influence memory versus effector cell differentiation [4], [5], [39]. Based on their expression pattern described here, BTLA and TIM-3 are important already for naive T-cells, not excluding an important function in differentiated cells, while PD-1, CTLA-4, KLRG-1, 2B4, LAG-3 and CD160 primarily modulate the functions of differentiated T-cells.

In comparison to total CD8 T-cells, non-naive cells specific for the tumor-antigen Melan-A show distinct expression patterns, essentially with more TIM-3 and PD-1 and less CD160 (*Figure 6*). The particularities of inhibitory receptor co-expression on Melan-A specific T-cells were induced after priming, as Melan-A-specific T-cells from healthy donors expressed inhibitory receptors highly similar to total naive T-cells. Potent priming, such as with CpG-ODN 7909 induced upregulation of KLRG-1 and TIM-3, and concomitant downregulation of BTLA [9] compared to less potent priming in the absence of the TLR-9 agonist CpG.

Confirming our previous report [1], CD8 T-cells specific for the viral-antigens BMLF1 (EBV) and pp65 (CMV) displayed a different pattern of inhibitory receptors compared to cells specific for tumor-antigens. Apart from their specificity, these differences between viral- and tumor-specific T-cells could also be due to their distinct genesis. Unlike EBV- and CMV-specific T-cells, Melan-A-specific T-cells studied were induced by vaccination, and vaccine components can have a direct impact on inhibitory receptor expression as we exemplified by the use of CpG-ODN. Since we could not test the influence of other adjuvants or vaccine formulations, it remains unknown whether the effect of CpG-ODN was particularly strong, which may well be the case given the unusually strong T-cell activation promoted by CpG-ODN [32]. For further characterization of vaccine-mediated effects, it would be interesting to study vaccine induced anti-viral immune responses. This could be done with yellow fever or small pox vaccination, which are known to induce robust CD8 T-cell responses [40], [41]. Since these vaccines use live attenuated viruses in contrast to the peptide vaccination used in our study, one may find different patterns. However, it is possible that high inhibitory receptor expression primarily depends on the capacity of vaccines to generate effector cells, which is high for CpG-ODN adjuvanted vaccines and for live vaccines.

Based on their high precursor frequencies [35], it has been argued that Melan-A-specific T-cells are unusual and not representative of other tumor-antigen-specific T-cells. However, cells specific for NY-ESO-1 and for MAGE-A10, two other tumor-antigen specific T-cell populations with lower precursor frequencies, were comparable to Melan-A-specific T-cells regarding the expression of inhibitory receptors, arguing that higher TIM-3 and PD-1 expression and lower CD160 expression is a general feature of self/tumor-specific T-cells.

Apart from antigen-specificity and the type of T-cell stimulation, the microenvironment was also found to strongly influence the expression of inhibitory receptors on CD8 T-cells. Tumor-specific T-cells from TILN were lower in KLRG-1 expression but higher in TIM-3, LAG-3 and CTLA-4 expression as compared to cells with the same specificity in blood (*Figure 6*). Analysis of EBV-specific T-cells in TILN revealed that the upregulation of LAG-3 and CTLA-4 (but not of TIM-3) and the downregulation of KLRG-1 were at least partially due to the microenvironment in the lymph node. To further investigate how much of this regulation is induced by the tumor cells present and how much is due to the microenvironment normally found in lymph nodes, experiments utilizing normal lymph nodes and non-lymphoid tumor metastasis or primary tumor samples are warranted.

Inhibition of T-cell functions depends on interactions of inhibitory receptors with their ligands. Therefore we analyzed whether the respective ligands are present in the tumor microenvironment. In the majority of patients we found multiple ligands, such as PD-L2, galectin-9, HVEM, MHC class II, CD86 and CD80, but not E-cadherin. The presence of these ligands within a metastatic lesion together with the upregulation of the inhibitory receptors on the tumor-specific CD8 T-cells fits well with the previously described functional deficiency [42] and the exhaustion profile exhibited by tumor-specific T-cells in metastatic lesions [1]. However, the expression of several ligands was primarily intracellular, leaving the questions open regarding eventual low-level surface expression and functional impact. Further studies are necessary to detail the exact contribution of each receptor-ligand interaction [43].

In summary, we found that inhibitory receptor co-expression on antigen-specific T-cells strongly depended on their differentiation status, antigen-specificity and on the tumor microenvironment. These data support the rational for therapeutic blocking of multiple inhibitory receptors, with the aim to increase the potential of antigen-specific T-cells to confer immunity to cancer and infection.

## Supporting Information

Figure S1 **Gating strategy, and inhibitory receptor expression in total CD8 T-cells.** (A) Gating strategy. Expression of inhibitory receptors was analyzed on total CD8+ T-cells, on naive, central memory, effector memory and effector memory RA+ cells (based on CCR7 and CD45RA expression) and on tetramer positive cells. Staining 1 and staining 2 each contained four antibodies specific for four different inhibitory receptors (upper histograms). Isotype controls were used as negative controls (lower histograms). (B) PBMCs were enriched for CD8 using magnetic beads. Naive (N), central memory (CM), effector memory (EM) and effector memory RA^+^ (EMRA) cells were defined by the expression of CCR7 and CD45RA. Positivity for the inhibitory receptor was defined respective to isotype controls. p-values represent the results of the one-way ANOVA test.
(TIF)

Figure S2 **Influence of priming on expression of inhibitory receptors.** Samples from healthy donors (HD) or patients before vaccination (before vacc.) or after peptide+IFA vaccination, either with or without CpG-ODN, were enriched for CD8 using magnetic beads. Melan-A-specific T-cells were identified using CD8-specific antibody and tetramer as described in the Materials and Methods section. Positivity for the inhibitory receptor was defined respective to isotype controls. p-values represent the results of the one-way ANOVA test.
(TIF)

Figure S3 **Expression of inhibitory receptors on tumor- and virus-specific CD8 T-cells.** Samples from blood from patients were enriched for CD8 T-cells using magnetic beads. Melan-A-, CMV- and EBV-specific CD8 T-cells were identified by staining with CD8-specific antibody and tetramers as described in the Materials and Methods section. Positivity for the inhibitory receptor was defined respective to isotype controls. n = 11/14/9 for Melan-A-, n = 7/8/3 for CMV- and n = 15/18/8 for EBV-specific T-cells (staining 1 / LAG3, 2B4 / BTLA, CTLA-4). (B) Hierarchical clustering based on co-expression of the eight inhibitory receptors shown in A, including the four differentiation subsets (N, CM, EM, EMRA) of total CD8 T-cells. (C) Principal Component Analysis based on the same data as in (B). Ellipses represent the 80-percent level of the population while the crosses indicate the mean of each population. Melan-A-specific cells are represented as black dots without the ellipse.
(TIF)

Figure S4 **Expression of inhibitory receptors on self/tumor-specific CD8 T-cells.** PBMC from four patients were enriched for CD8 T-cells using magnetic beads. Melan-A-, NY-ESO-1- and MAGE-A10-specific CD8 T-cells were identified by staining with CD8-specific antibody and tetramers as described in the Materials and Methods section. Positivity for the inhibitory receptor was defined respective to isotype controls.
(TIF)

Figure S5 **Influence of the microenvironment on expression of inhibitory receptors.** Samples from patients vaccinated either with (red) or without (black) CpG-ODN were enriched for CD8 using magnetic beads. Melan-A-specific T-cells were identified using CD8-specific antibody and tetramer as described in the Materials and Methods section. Positivity for the inhibitory receptor was defined respective to isotype controls.
(TIF)

Table S1 **Inhibitory receptors and identified ligands.** For each of the eight inhibitory receptors investigated the known ligands are listed.
(PDF)
