# Supplementary figures and images for: Extended Co-Expression of Inhibitory Receptors by Human CD8 T-Cells Depending on Differentiation, Antigen-Specificity and Anatomical Localization
Source: PLoS One. 2012 Feb 8;7(2):e30852. doi: 10.1371/journal.pone.0030852 (PMC3275569; doi:10.1371/journal.pone.0030852)

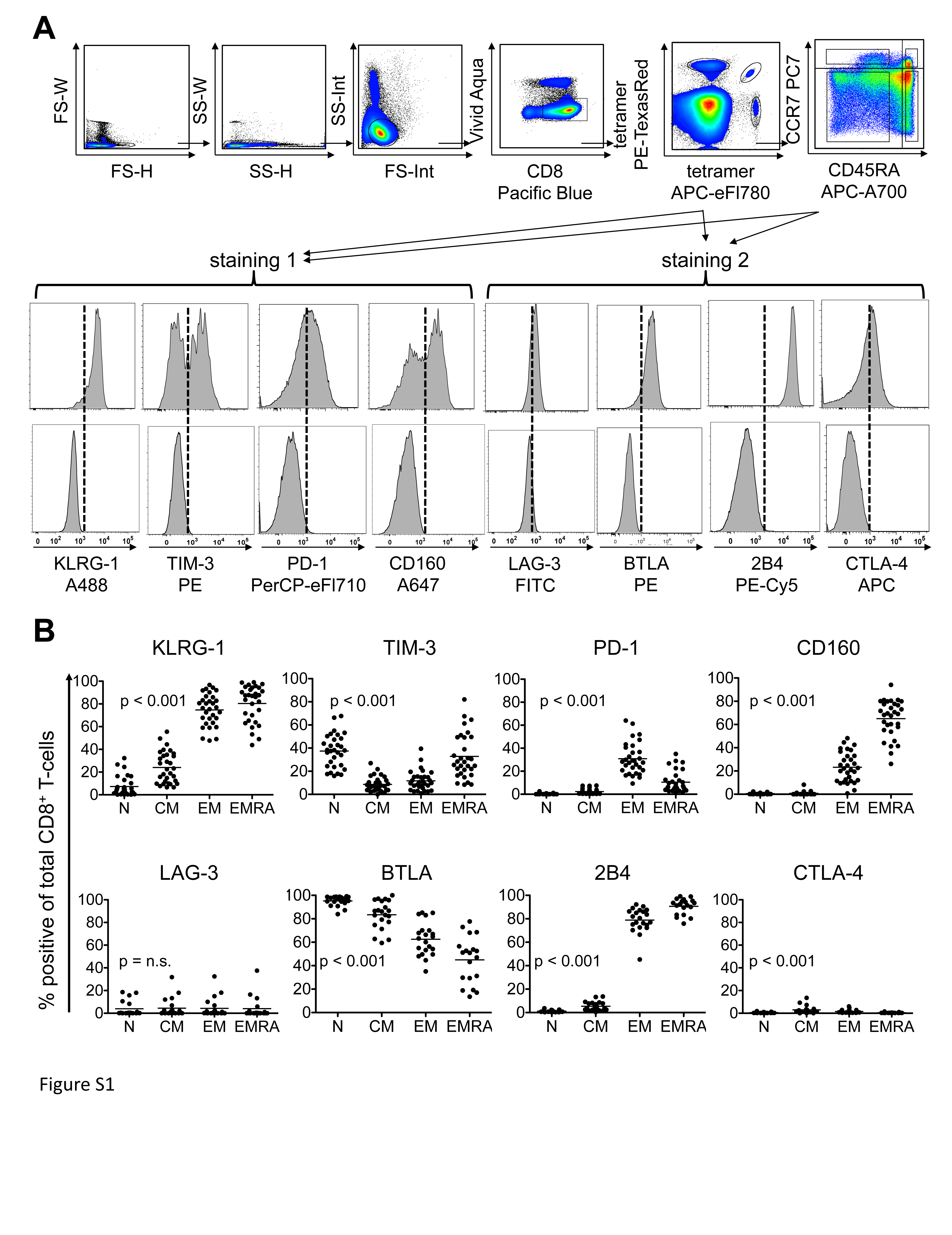

Supplement: Figure S1 — Gating strategy, and inhibitory receptor expression in total CD8 T-cells. (A) Gating strategy. Expression of inhibitory receptors was analyzed on total CD8+ T-cells, on naive, central memory, effector memory and effector memory RA+ cells (based on CCR7 and CD45RA expression) and on tetramer positive cells. Staining 1 and staining 2 each contained four antibodies specific for four different inhibitory receptors (upper histograms). Isotype controls were used as negative controls (lower histograms). (B) PBMCs were enriched for CD8 using magnetic beads. Naive (N), central memory (CM), effector memory (EM) and effector memory RA+ (EMRA) cells were defined by the expression of CCR7 and CD45RA. Positivity for the inhibitory receptor was defined respective to isotype controls. p-values represent the results of the one-way ANOVA test. (TIF) [file pone.0030852.s001.tif]

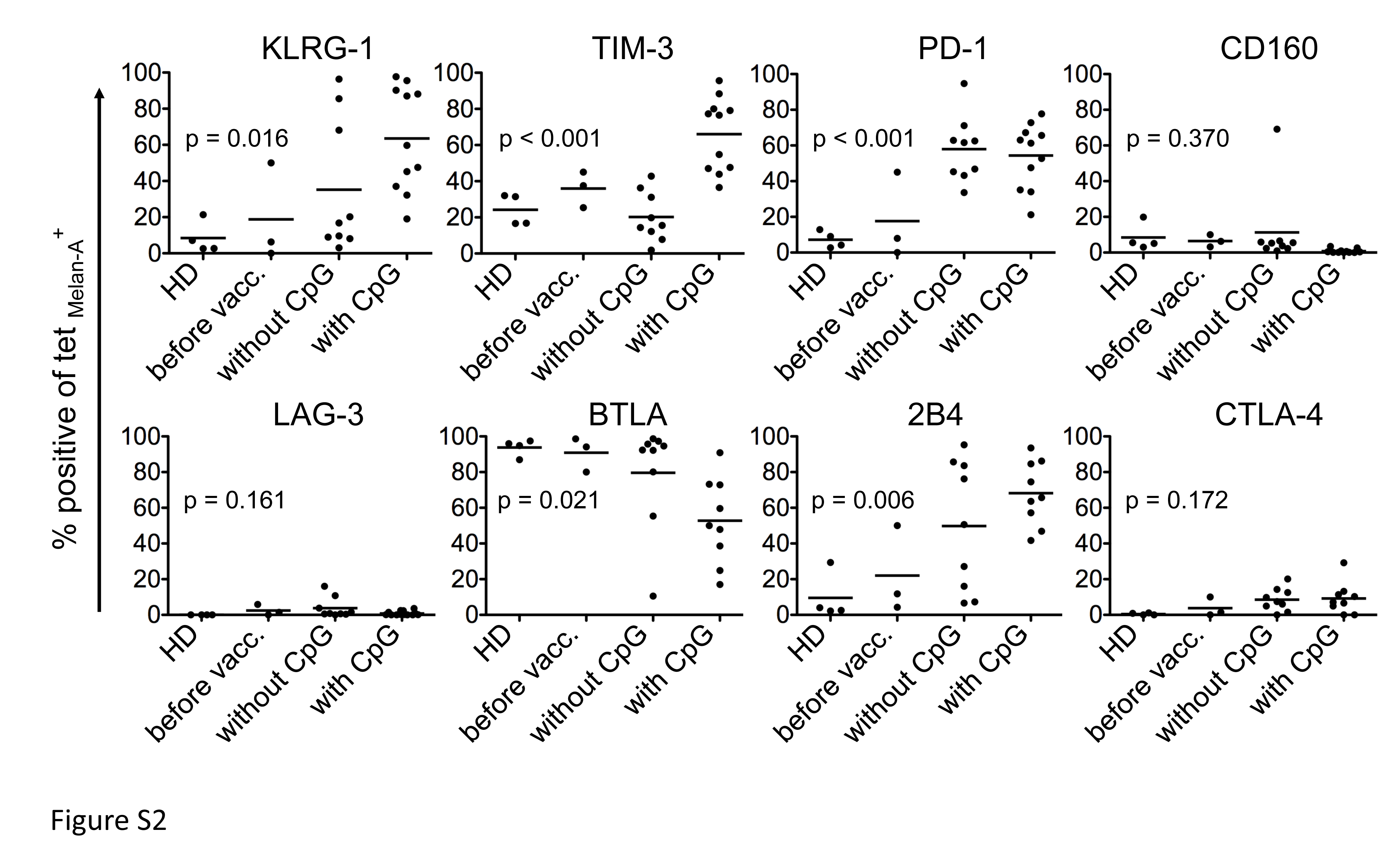

Supplement: Figure S2 — Influence of priming on expression of inhibitory receptors. Samples from healthy donors (HD) or patients before vaccination (before vacc.) or after peptide+IFA vaccination, either with or without CpG-ODN, were enriched for CD8 using magnetic beads. Melan-A-specific T-cells were identified using CD8-specific antibody and tetramer as described in the Materials and Methods section. Positivity for the inhibitory receptor was defined respective to isotype controls. p-values represent the results of the one-way ANOVA test. (TIF) [file pone.0030852.s002.tif]

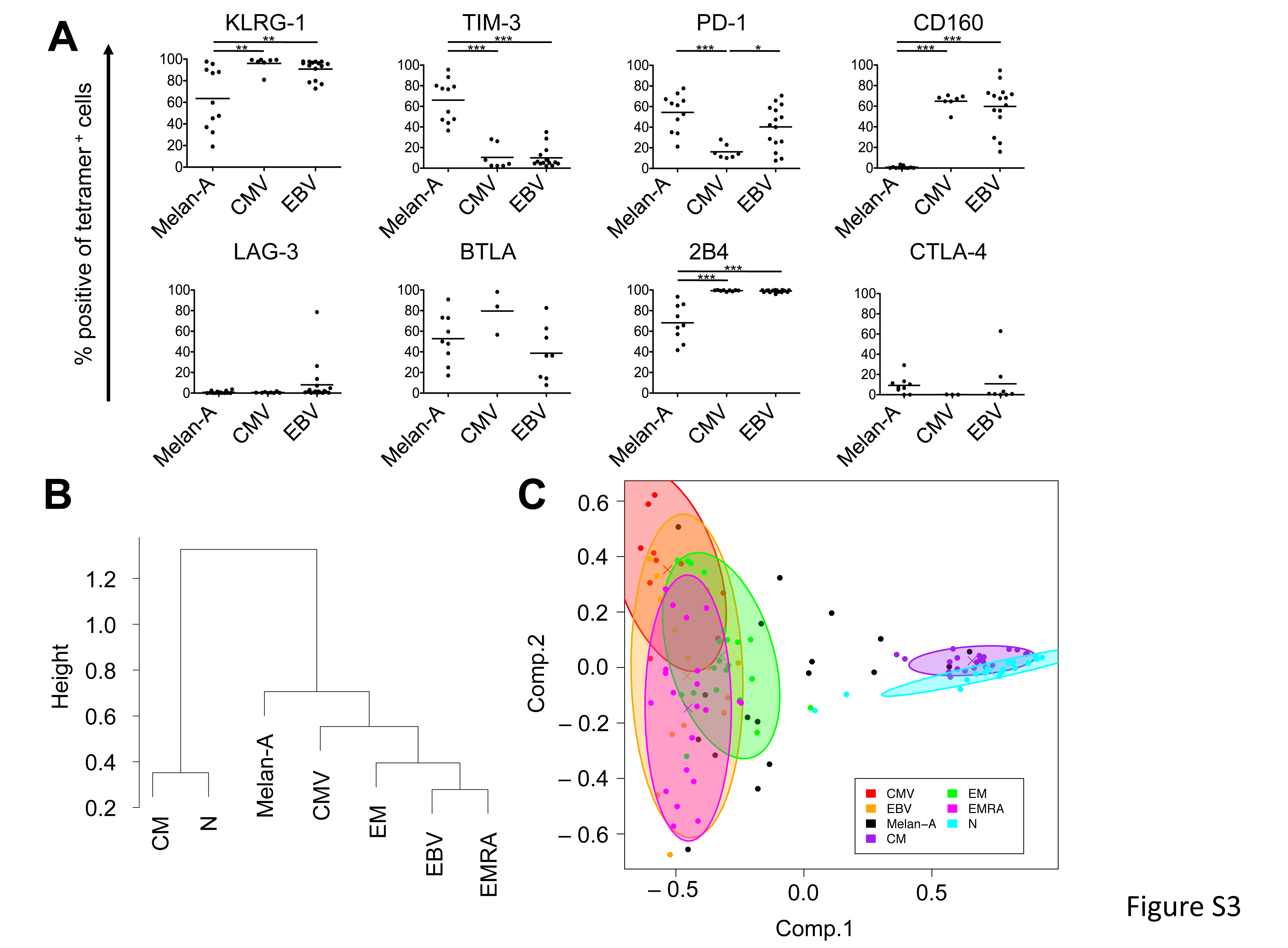

Supplement: Figure S3 — Expression of inhibitory receptors on tumor- and virus-specific CD8 T-cells. Samples from blood from patients were enriched for CD8 T-cells using magnetic beads. Melan-A-, CMV- and EBV-specific CD8 T-cells were identified by staining with CD8-specific antibody and tetramers as described in the Materials and Methods section. Positivity for the inhibitory receptor was defined respective to isotype controls. n = 11/14/9 for Melan-A-, n = 7/8/3 for CMV- and n = 15/18/8 for EBV-specific T-cells (staining 1 / LAG3, 2B4 / BTLA, CTLA-4). (B) Hierarchical clustering based on co-expression of the eight inhibitory receptors shown in A, including the four differentiation subsets (N, CM, EM, EMRA) of total CD8 T-cells. (C) Principal Component Analysis based on the same data as in (B). Ellipses represent the 80-percent level of the population while the crosses indicate the mean of each population. Melan-A-specific cells are represented as black dots without the ellipse. (TIF) [file pone.0030852.s003.tif]

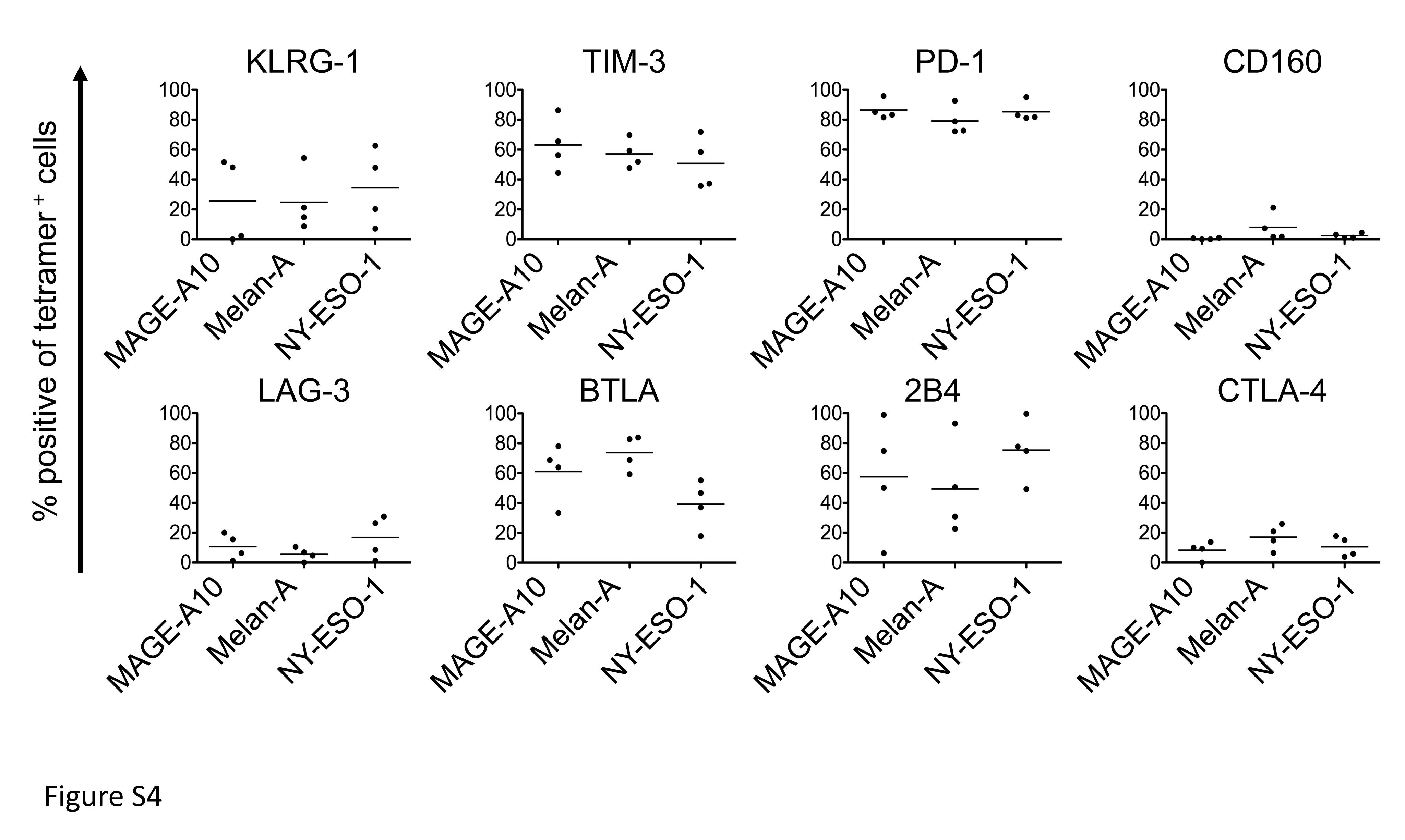

Supplement: Figure S4 — Expression of inhibitory receptors on self/tumor-specific CD8 T-cells. PBMC from four patients were enriched for CD8 T-cells using magnetic beads. Melan-A-, NY-ESO-1- and MAGE-A10-specific CD8 T-cells were identified by staining with CD8-specific antibody and tetramers as described in the Materials and Methods section. Positivity for the inhibitory receptor was defined respective to isotype controls. (TIF) [file pone.0030852.s004.tif]

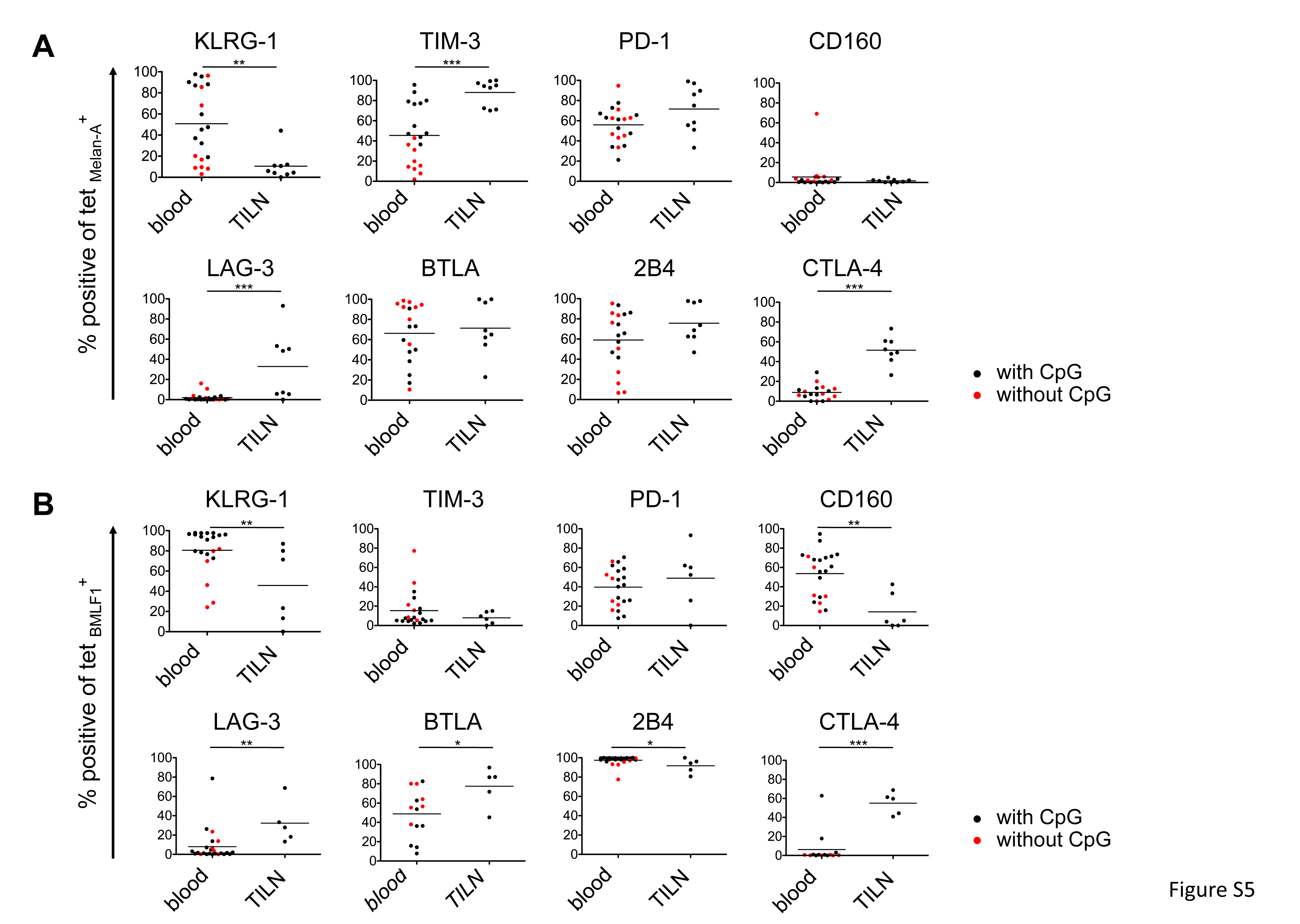

Supplement: Figure S5 — Influence of the microenvironment on expression of inhibitory receptors. Samples from patients vaccinated either with (red) or without (black) CpG-ODN were enriched for CD8 using magnetic beads. Melan-A-specific T-cells were identified using CD8-specific antibody and tetramer as described in the Materials and Methods section. Positivity for the inhibitory receptor was defined respective to isotype controls. (TIF) [file pone.0030852.s005.tif]
