# Supplementary material for: Extended Co-Expression of Inhibitory Receptors by Human CD8 T-Cells Depending on Differentiation, Antigen-Specificity and Anatomical Localization
Source: PLoS One. 2012 Feb 8;7(2):e30852. doi: 10.1371/journal.pone.0030852 (PMC3275569; doi:10.1371/journal.pone.0030852)
Supplement: Table S1 — Inhibitory receptors and identified ligands. For each of the eight inhibitory receptors investigated the known ligands are listed. (PDF) [file pone.0030852.s006.pdf]

Supplementary Tables

Supplementary Table 1 Inhibitory receptors and their respective ligands

**inhibitory receptor**	**ligand**	**inhibitory receptor**	**ligand**
KLRG-1	E-cadherin	LAG-3	MHC-II
TIM-3	galectin-9	BTLA	HVEM
PD-1	PD-L1 / PD-L2	2B4	CD48
CD160	HVEM	CTLA-4	CD80 / CD86
